# Head Nurses' Leadership Styles and Emergency Department Nurses' Clinical Professional Autonomy in Iranian University Hospitals: A Cross-Sectional Study

**DOI:** 10.1155/jonm/1376035

**Published:** 2025-11-29

**Authors:** Mina Omidvar, Roohangiz Norouzinia, Maryam Aghabarary, Pardis Rahmatpour

**Affiliations:** ^1^Student Research Committee, Alborz University of Medical Sciences, Karaj, Iran; ^2^Social Determinants of Health Research Center, Alborz University of Medical Sciences, Karaj, Iran; ^3^Health Sciences Research Unit: Nursing (UICISA: E), Nursing School of Coimbra (ESEnfC), Coimbra, Portugal

**Keywords:** clinical professional autonomy, emergency nurses, leadership, nurse managers

## Abstract

**Introduction:**

Nurses' clinical professional autonomy is strongly influenced by organizational management and leadership environments. This study aims to examine the relationship between head nurses' leadership styles and the level of clinical autonomy perceived by emergency department (ED) nurses in Iranian university hospitals.

**Methods:**

This cross-sectional study recruited 221 ED nurses from Alborz province (Iran) university hospitals through convenience sampling from February to March 2022. Data were collected using a three-section questionnaire that covered demographic and work-related characteristics, the Multifactor Leadership Questionnaire (MLQ), and the Clinical Professional Autonomy Scale. Following psychometric validation in our sample, the MLQ consisted of 32 items assessing transformational, transactional, and laissez-faire leadership (Cronbach's *α* = 0.71–0.95). Clinical professional autonomy was measured with the 31-item Clinical Professional Autonomy Scale (*α* > 0.70), with total scores classified as low, moderate, or high.

**Results:**

The mean (SD) of clinical professional autonomy (0–100 scale) was 78.68 (12.73). Transformational leadership was reported as the dominant style among head nurses (72.41 ± 18.58). The results showed the strongest correlation between transformational leadership style and clinical professional autonomy (*r* = 0.57). Furthermore, the multivariable linear regression analysis showed that clinical professional autonomy was significantly associated with job satisfaction (*β* = 0.17, *p*=0.026) and nurses' years of work experience (*β* = 0.23, *p*=0.002).

**Conclusion:**

This study highlights that head nurses' transformational leadership style significantly enhances nurses' clinical professional autonomy. The findings underscore the need for targeted leadership development programs to strengthen head nurses' transformational leadership competencies. By fostering a supportive leadership environment, clinical professional nursing autonomy can be promoted, contributing to improved patient care quality and overall organizational performance.

## 1. Introduction

Head nurses in emergency departments (EDs) play a critical role in shaping clinical environments that promote professional development and support safe, autonomous nursing practice. Among the core values of the nursing profession, clinical autonomy—defined as the ability of nurses to make independent decisions based on their clinical judgment and ethical principles—is essential for ensuring high-quality care, especially in complex and high-pressure settings such as EDs [1, 2]. Effective leadership not only enhances operational efficiency but also shapes the extent to which nurses can exercise professional autonomy [3]. Prior research indicates that managerial approaches and supervisory styles have a measurable impact on nursing care quality, reinforcing the need for strong and supportive leadership in clinical settings [4]. Approaches that empower nurses and safeguard their autonomy are particularly vital in EDs, where rapid decision-making and independent judgment are essential [3, 5]. The style of management adopted by head nurses can either foster or hinder autonomy, thereby affecting job performance, innovation, and ultimately patient outcomes [6, 7].

A recent study has underscored the relevance of leadership to various nursing outcomes. Evidence suggests that head nurse empowerment enhances nurses' innovative behavior through pathways involving professional autonomy and organizational climate [6]. Furthermore, professional autonomy has been shown to improve job performance directly and indirectly by increasing job satisfaction and organizational commitment [8]. In addition, findings from Oman indicate that authentic leadership among nurse managers is independently associated with lower burnout levels among emergency nurses, highlighting the critical role of leadership style in promoting staff well-being in high-stress clinical environments [9].

Despite the increasing attention to leadership within nursing practice, limited research has specifically examined how various leadership styles adopted by head nurses predict professional autonomy among ED nurses, particularly in the context of Iranian university hospitals. The majority of existing studies have focused on related variables such as burnout, job satisfaction, or moral distress [10, 11], with relatively little empirical investigation into the predictive role of leadership style in shaping nurses' autonomy in this unique clinical setting.

Building on these findings, the present study investigates the relationship between head nurses' leadership styles and the clinical professional autonomy of nurses working in EDs within Iranian university hospitals. The findings from this study may provide evidence-based insights for nursing managers and policymakers to promote clinical professional autonomy, support workforce sustainability, and improve the quality of emergency care.

## 2. Methods

### 2.1. Study Design

A cross-sectional study was conducted among ED nurses working in university hospitals in Alborz Province (Iran) between February and March 2022.

### 2.2. Study Participants

Participants were selected through convenience sampling based on specific eligibility criteria. Inclusion criteria were willingness to participate in the study, full-time employment in the ED, and a minimum of 6 months of work experience in the ED. Participants who submitted questionnaires with more than 10% incomplete responses were excluded. Out of 276 eligible nurses, 221 completed the study (response rate = 80%).

### 2.3. Instruments

A structured questionnaire comprising three sections was distributed to the eligible nurses:

1. Demographic and work-related information: this section included variables such as age, gender, years of work experience in the ED, job satisfaction, and willingness to transfer from the ED.
2. The Multifactor Leadership Questionnaire (MLQ): this scale is one of the standard tools used to measure leadership styles in organizations [12]. It has been widely used in studies to evaluate different aspects of leadership. It consists of 36 items that ask nurses to respond about the leadership style of their supervisors in terms of transformational (*n* = 20), transactional (*n* = 12), and laissez-faire leadership styles (*n* = 4), with a five-point Likert scale from “always” (5) to “never” (1) [13]. At the time of writing this article, the psychometric properties of the 36-item questionnaire had not been evaluated among the Iranian nursing population. Before conducting data analysis, we conducted an exploratory factor analysis (EFA) employing maximum likelihood and Promax rotation to assess the questionnaire in the current study population (KMO = 0.93; Bartlett's test of sphericity: 5066 (df: 496), *p* < 0.001; and total explained variance = 54.4%). Therefore, based on the EFA results, four items (2, 7, 13, and 22) were removed due to factor loadings below 0.3. The final version included 32 items across the three dimensions: Transformational (19 items; factor loading range = 0.53–0.87), transactional (10 items; factor loading range = 0.67–0.91), and laissez-faire leadership styles (3 items; factor loading range = 0.51–0.69). Subscale scores were calculated separately, transformed to 0–100 using the formula: (observed score − minimum score) ÷ (maximum score − minimum score) × 100. Higher scores indicate a more dominant leadership style. The Cronbach's alpha values for transformational, transactional, and laissez-faire leadership styles were 0.95, 0.87, and 0.71, respectively, and a Cronbach's alpha ≥ 0.70 was considered acceptable [14]. This indicates that the reliability of the leadership styles has been confirmed.
3. The Clinical Professional Autonomy Scale: this scale was developed and validated in Iran [2]. It comprises 31 items rated on a Likert scale ranging from always (5) to never (1). Final scores were converted to a 0–100 scale (similar to MLQ), where 0 indicates no professional autonomy, scores ≤ 33.3 represent low autonomy, scores from 33.4 to 66.6 indicate moderate autonomy, and scores ≥ 66.7 reflect high autonomy.

### 2.4. Data Analysis

Descriptive statistics (frequency and percentage, mean and standard deviation) and inferential statistics (Pearson correlation and multivariate linear regression) were used to analyze the data by using SPSS v. 22. Before analysis, the normality of the variables was assessed through skewness (±3) and kurtosis (±7) [15]. A significance level of *p* ≤ 0.05 was considered for all statistical tests.

### 2.5. Ethical Considerations

Participation in the study was completely voluntary. Before distributing the questionnaires, the purpose and goals of the research were clearly explained to all eligible nurses. Questionnaires were given only to those who willingly agreed to participate. Participants were assured that all data would be kept strictly confidential, analyzed anonymously, and used only for research purposes. They were also informed of their right to withdraw from the study at any time without any consequences. Completing and returning the questionnaire was considered implied informed consent. The project of this study protocol was approved by the Ethics Committee of Alborz University of Medical Sciences (Code: IR.ABZUMS.REC.1400.224).

## 3. Results

Among the 221 participants, 159 (71.9%) were female and 143 (64.7%) were married. The mean age of the nurses was 30.59 ± 4.44 years (range: 23–49 years), and their mean work experience was 7.36 years (SD = 4.61 and range: 6 months–28 years). The majority of nurses (62.4%) reported moderate job satisfaction. Additionally, 107 participants (48.4%) reported a low willingness to transfer from the ED.

The mean score of clinical professional autonomy was 78.68 ± 12.73, indicating a high level of clinical autonomy according to the scale categorization. The mean and standard deviation scores for leadership styles were as follows: transformational leadership, 72.41 ± 18.58; transactional leadership, 72.24 ± 16.61; and laissez-faire leadership, 47.55 ± 24.62. These results indicate that the dominant leadership style among head nurses was transformational leadership (Table 1).

Furthermore, the Pearson correlation test revealed that all three leadership styles had a significant positive correlation with clinical professional autonomy. Among them, transformational leadership showed the strongest association (*r* = 0.57), followed by transactional leadership (*r* = 0.55), and finally, laissez-faire leadership (*r* = 0.15) (Table 1).

Among the demographic and work-related variables included in the multivariate linear regression model, job satisfaction (*B* = 3.04, SE = 1.36, 95% CI [0.02, 0.32], *β* = 0.17, and *p*=0.026) and nurses' years of work experience (*B* = 0.04, SE = 0.01, 95% CI [0.08, 0.37], *β* = 0.23, and *p*=0.002) showed significant positive associations with clinical professional autonomy (see Table 2).

## 4. Discussion

The present study identified transformational leadership as the predominant style among head nurses in EDs, a finding consistent with existing literature on leadership in high-stress clinical settings. Previous research has demonstrated that transformational leadership in healthcare is associated with improved nurse outcomes, including enhanced job satisfaction [16, 17], increased structural empowerment [18], and reduced adverse patient events [17]. For example, Labrague reported that transformational leadership in emergency care improves care quality and decreases clinical errors, largely by elevating nurse job satisfaction [17]. Similarly, Niinihuhta et al. emphasized that transformational leadership supports nurse leaders' well-being and fosters an empowering organizational climate [18].

These advantages are particularly important in the unpredictable and high-pressure context of EDs, as they may contribute to strengthening nurses' clinical professional autonomy, promoting innovation, and enhancing confidence in clinical decision-making. These findings align with transformational leadership theory, originally conceptualized by Burns and further elaborated by Bass and Avolio [12], which identifies core leader behaviors—idealized influence, inspirational motivation, intellectual stimulation, and individualized consideration—that inspire followers to exceed expectations and foster psychological empowerment and autonomy [19]. Furthermore, self-determination theory [20] provides a complementary explanatory framework by positing autonomy, competence, and relatedness as essential psychological needs; recent healthcare research indicates that transformational leadership can fulfill these needs, thereby enhancing nurses' intrinsic motivation and perceived autonomy in practice settings [21]. Together, these theoretical perspectives deepen the conceptual clarity of our findings, suggesting mechanisms through which transformational leadership may shape clinical professional autonomy and motivation among ED nurses.

The finding that ED nurses reported high levels of clinical professional autonomy is partially consistent with previous Iranian research. While some national studies, such as Balasi et al., have indicated high levels of autonomy among clinical nurses [5], and others conducted in critical care and specialized units have reported only moderate levels of autonomy [8, 22]. Likewise, international findings on this issue remain mixed. For instance, Pursio et al. reported autonomy levels above the mid-range among nurses [23], while studies by Alruwaili et al. conducted in acute care settings, found moderate autonomy levels [24, 25]. It is plausible that professional competence in emergency and intensive care settings develops progressively, due to frequent exposure to acute situations that demand independent nursing interventions. These experiences reinforce clinical expertise and build confidence in autonomous practice [5]. Furthermore, the timing of data collection—during the COVID-19 pandemic—may have contributed to higher reported clinical autonomy, as emergency nurses likely experienced greater freedom in clinical decision-making. Another important factor is the use of a culturally adapted measurement instrument specifically designed for the Iranian nursing context. This may account for discrepancies between the present findings and those of other studies, as instruments developed in other countries may not fully capture the nuanced dimensions of clinical professional autonomy within distinct cultural and healthcare system frameworks.

The study also identified a significant positive correlation between head nurses' leadership styles and the clinical professional autonomy of nurses working in EDs. Among the leadership styles examined, transformational leadership showed the strongest association with clinical professional autonomy, both in terms of intensity and impact. Transactional leadership followed, while the laissez-faire style also demonstrated a positive, though comparatively weaker, correlation with nurses' clinical professional autonomy. These results are consistent with earlier studies that have emphasized the positive impact of transformational leadership on nurses' autonomy and decision-making capacity [26, 27]. Pursio et al. further noted that effective nursing leadership, active clinical involvement, knowledge sharing, interprofessional collaboration, and adherence to quality standards are all associated with enhanced professional autonomy. Pursio's study also highlighted a strong correlation between autonomy and job satisfaction [23]. Similarly, Alruwaili and Abuadas pointed out that in Saudi Arabia, cultural and hierarchical factors can constrain nurses' autonomy, particularly in administrative or policy-related roles [24]. They recommended enhancing autonomy through leadership development programs, institutional support mechanisms, and formal recognition of nurses' clinical judgment and decision-making authority.

Although no Iranian studies to date have directly examined the relationship between leadership styles and clinical professional autonomy, some qualitative investigations suggest an indirect link. For instance, Setoodegan et al. described how managerial support and involvement in decision-making processes influence nurses' experiences of autonomy [28]. Likewise, AllahBakhshian et al. reported that authoritarian leadership styles and ambiguous role definitions limited ICU nurses' capacity for independent action [29]. Although leadership style was not the primary focus in these studies, the findings imply that leadership dynamics may play a crucial role in shaping professional autonomy within clinical settings.

Moreover, this study found a significant positive correlation between nurses' job satisfaction and their perceived clinical professional autonomy. These results align with those of Mahoney et al., who underscored the critical role of professional autonomy in enhancing job satisfaction. The present findings further suggest that high levels of autonomy and job satisfaction can jointly reduce job burnout, reinforce organizational commitment, and lower nurses' intent to leave the profession [30]. In support of this, Pursio et al. reported a strong association between autonomy and job satisfaction, reinforcing the value of autonomy-supportive environments in high-performing hospitals [23]. In the same context, Judi et al. provided empirical evidence showing that professional autonomy not only directly improves nurse performance but also does so indirectly by increasing job satisfaction and commitment [8].

The present study demonstrated that nurses' clinical work experience is positively associated with professional autonomy. This finding aligns with prior research indicating that accumulated experience enhances clinical judgment, confidence, and decision-making skills—key components of autonomous practice. For example, Balasi et al. found that nurses with more years of experience achieved higher autonomy scores, indicating that hands-on practice strengthens the ability to make independent clinical decisions [5, 10, 31]. Similarly, Pursio et al. emphasized that professional competence and tenure contribute to nurses' autonomy, particularly among senior or managerial staff [1]. Collectively, these studies support the idea that strategies aiming to foster professional autonomy should consider nurses' work experience, while also providing mentorship to less-experienced staff to accelerate their development.

Overall, these converging findings underscore the critical role of clinical professional autonomy in enhancing nurse job satisfaction, performance, and retention—particularly in high-pressure environments such as EDs. Moreover, nurses with greater clinical experience tend to exhibit higher levels of autonomy. Leadership styles, especially transformational leadership, significantly influence the development of autonomy, highlighting the importance of leadership training and supportive organizational cultures that empower nurses to fully exercise their professional roles.

### 4.1. Study Limitations

Several limitations should be considered when interpreting these findings. First, the sample was restricted to nurses from a single province in Iran, limiting generalizability to other cultural and organizational contexts. Future studies should therefore consider multicenter or national-level sampling to capture a wider diversity of institutional cultures and nurse demographics. Second, reliance on self-reported questionnaires may have introduced response bias, particularly social desirability bias. Third, the gender imbalance in the sample could have affected perceptions of leadership and autonomy. Fourth, data collection during the COVID-19 pandemic—characterized by increased workload and stress—may have temporarily influenced nurses' experiences and perceptions.

In terms of measurement tools, while the Nurses' Clinical Professional Autonomy Questionnaire was culturally adapted and validated, MLQ, originally developed in a different cultural context, may still carry subtle biases. Future studies should conduct confirmatory factor analysis of the MLQ in Iranian and other Middle Eastern nursing populations to further establish its cultural applicability.

Additionally, potential confounders such as shift type, hospital type, and patient acuity were not controlled for in this study. Future research should account for these contextual variables and also explore causal relationships more explicitly. In this regard, adopting longitudinal designs would help track changes in leadership practices and professional autonomy over time, thereby clarifying causal pathways and the sustainability of observed effects. Likewise, a mixed-methods approach—combining quantitative surveys with qualitative interviews—could provide deeper insights into how leadership dynamics are perceived and enacted in EDs. Finally, future studies should also examine the potential mediating and moderating roles of factors such as job satisfaction, burnout, and organizational culture in the relationship between leadership style and professional autonomy.

### 4.2. Implications for Future Research, Nursing Practice, Education, and Policy

#### 4.2.1. Future Research

Future studies should consider multicenter or longitudinal designs and examine potential mediators or moderators (e.g., burnout, resilience, and organizational culture) to strengthen the generalizability and depth of understanding.

#### 4.2.2. For Nursing Education

The positive association between transformational leadership and professional autonomy underscores the importance of integrating leadership training into educational and professional development programs for nurse managers. Training should emphasize transformational competencies such as individualized consideration, intellectual stimulation, and inspirational motivation, which are the key to fostering environments that support nurses' autonomy and professional growth.

#### 4.2.3. For Nursing Management Practice

Healthcare institutions should adopt management strategies that promote autonomy-supportive cultures. This includes encouraging shared governance structures, decentralization of decision-making, and recognition systems that validate nurses' clinical judgment. Monitoring autonomy-related factors through staff evaluations and satisfaction surveys can provide actionable feedback for improving job satisfaction and retention.

#### 4.2.4. For Policy and Administration

At the policy level, healthcare organizations and nursing policymakers should develop and implement policies that formalize autonomy-supportive leadership as a benchmark for quality assurance, particularly in emergency and critical care settings. Establishing supportive structures—such as clear role definitions, recognition of nurses' decision-making authority, and integration of leadership behavior assessments into performance evaluation frameworks—can further strengthen professional autonomy at the organizational level.

## 5. Conclusion

This study highlights a significant positive correlation between head nurses' leadership styles and the clinical professional autonomy of ED nurses, with transformational leadership showing the strongest association. Professional autonomy was also positively associated with job satisfaction. These findings suggest that both leadership style and job satisfaction play essential roles in fostering nurses' autonomy. Healthcare organizations may benefit from leadership development programs focused on transformational competencies—such as mentoring, inspirational motivation, and individualized consideration—to strengthen autonomy in nursing practice. Additionally, incorporating performance appraisal systems that assess transformational leadership behaviors could further promote and sustain leadership practices that support professional autonomy in clinical settings.

## Figures and Tables

**Table 1:** Descriptive statistics and correlations among leadership styles and professional autonomy.

Study variables	Frequency summary				Correlation matrix			
	Mean	SD	Skewness	Kurtosis	1	2	3	4
1. Transformational leadership	72.41	18.58	−0.70	0.18	1			
2. Transactional leadership	72.25	16.61	−0.58	0.36	0.84^∗^	1		
3. Laissez-faire leadership	47.55	24.62	0.22	−0.42	0.10	0.32^∗∗^	1	
4. Professional autonomy	78.68	12.73	−1.08	1.70	0.57^∗∗^	0.55^∗∗^	0.15^∗^	1

^∗^ *p* ≤ 0.05.

^∗∗^ *p* ≤ 0.01.

**Table 2:** Multivariate linear regression of demographic and work-related variables predicting clinical professional autonomy.

Variables	Beta	SE	*p*	Stand. beta	95% CI		VIF	Tolerance
					Lower	Upper		
Sex	−2.17	1.75	0.21	−0.09	−0.23	0.05	1.03	0.97
Work experience	0.04	0.01	0.002	0.23	0.08	0.37	1.05	0.95
Willingness to transfer	0.75	1.23	0.543	0.04	−0.10	0.19	1.14	0.87
Job satisfaction	3.04	1.36	0.026	0.17	0.02	0.32	1.17	0.85

*Note:* Model *R*^2^ = 10.4%.

Abbreviations: CI, confidence interval; VIF, variance inflation factor.

## Data Availability

The data that support the findings of this study are available on request from the corresponding author.
